# Association between Human T-Cell Lymphotropic Virus Type 1 and 2 (HTLV 1/2) infection and tuberculosis: systematic review and meta-analysis

**DOI:** 10.1186/1742-4690-8-S1-A80

**Published:** 2011-06-06

**Authors:** Sérgio Arruda, Camila Loureiro, Marcos Almeida, Dayana Mendes, Maria F R Grassi, José R Lapa, Afrânio Kritski, Kristien Verdonck, Eduardo Gotuzzo, Bernardo Galvão-Castro

**Affiliations:** 1 Escola Bahiana de Medicina e Saúde Pública, Salvador, Bahia, Brazil; 2 Laboratório Avançado de Saúde Pública, Centro de Pesquisas Gonçalo Moniz, Fiocruz, Salvador, Bahia, Brazil; 3 Universidade Federal do Rio de Janeiro,Faculdade de Medicina, RJ, Brazil; 4 Instituto de Medicina Tropical Alexander von Humboldt, Universidad Peruana Cayetano Heredia, Lima, Peru; 5 Institute of Tropical Medicine, Antwerp, Belgium

## Background

HTLV-1 infection alters the immune function and increases the risk of several infectious diseases. In this meta-analysis, we assess the association between HTLV- 1/2 and active tuberculosis (TB).

## Methods

Four databases were searched for relevant articles that describe the frequency of HTLV- 1/2 infection among TB patients and control groups of healthy individuals or patients without a history of TB. Data were analyzed using the EasyMA software.

## Results

The search yielded two hundred and eight articles. Six met the inclusion criteria and were included in the meta-analysis. The estimated relative risk of HTLV- 1/2 infection in TB patients was 3.25 times higher than in the population based control groups.

## Conclusion

Patients with active TB have a higher risk of HTLV- 1/2 infection. Prospective studies involving latent tuberculosis infection (LTBI) in HTLV-1-infected individuals are necessary to evaluate the potential benefit of TB chemoprophylaxis.

